# Production and downstream processing of (1→3)-β-D-glucan from mutant strain of *Agrobacterium sp*. ATCC 31750

**DOI:** 10.1186/2191-0855-2-31

**Published:** 2012-06-09

**Authors:** Gayathiri T Kalyanasundaram, Mukesh Doble, Sathyanarayana N Gummadi

**Affiliations:** 1 Department of Biotechnology, Applied and Industrial Microbiology Laboratory IIT Madras, Chennai, 600 036, India

**Keywords:** Curdlan, *Agrobacterium* sp. ATCC 31750, Chemical mutagenesis, Two stage culture, Downstream processing, Characterization

## Abstract

We isolated a mutant that produced higher levels of curdlan than the wild strain *Agrobacterium* sp. ATCC 31750 by chemical mutagenesis using N-methyl-N-nitro-nitrosoguanidine. The mutant strain produced 66 g/L of curdlan in 120 h with a yield of (0.88) while, the wild strain produced 41 g/L in 120 h with a yield of (0.62) in a stirred bioreactor. The mutant could not produce curdlan when the pH was shifted from 7.0 to 5.5 after nitrogen depletion as followed for wild strain. In contrast, pH optimum for cell growth and curdlan production for mutant was found to be 7.0. We optimized the downstream processing of curdlan by varying different volumes of NaOH and HCl for extraction and precipitation of curdlan. The molecular weight of the purified curdlan from the wild and mutant strain was 6.6 × 10^5^ Da and 5.8 × 10^5^ Da respectively. The monosaccharide analyses confirm that curdlan from both wild and mutant strain contains only glucose units. From the NMR and FTIR data, it has been confirmed that curdlan was exclusively composed of β (1 → 3)-D-glucan residues.

## Introduction

Curdlan is a high molecular weight, water insoluble (alkali soluble) extracellular polysaccharide composed only of β-(1→3) glucose residues (Lee et al. 1999). It acts as a structural macromolecule in the cell wall of yeast, mushrooms and other higher plants (Ko and Lin 2004). Curdlan is a secondary metabolite synthesized by *Alcaligenes faecalis* var. *myxogenes* and *Agrobacterium radiobacter* under nitrogen-limiting conditions (Wu et al. 2008& Lee et al. 1997). Since its discovery by Harada et. al. 1966, curdlan production has drawn considerable interest because of its unique rheological and thermal gelling properties. Curdlan is one of the FDA approved biopolymer used in food industries such as jelly, noodles, edible fibers manufacturing process. Curdlan is extensively used as an ingredient in animal feed since it acts as immune stimulator (Lee et al. 1999& Kumari and Sahoo 2006). It is used as concrete admixture and increases the water absorbing capacity of the concrete (Lee et al. 1997& Kim et al. 2000). It is also used as an immobilization support as it can covalently link available amino, hydroxyl and sulfhydryl groups of enzymes (Lee et al. 1999). Curdlan sulphate is developed as an antiviral agent against human immunodeficiency virus infections (Lee and Park 2001& Zhang et al. 2012). Curdlan stimulates nuclear factor kappa-B in macrophages and the activity is greatly enhanced by pre-treatment with sodium hydroxide or dimethyl sulfoxide (Kataoka et al. 2002).

Many studies have focused on optimizing several key factors including temperature, pH, agitation, aeration and nutrients involved in curdlan fermentation process to increase the yield. Previously curdlan production was also studied by using reactors with low shear system using axial flow marine type propeller that produced 46 g/l curdlan (Lee et al. 1999& Kim et al. 2000). During batch fermentation process, optimum pH for growth was 7.0 and pH was shifted to 5.5 after nitrogen depletion (Lee et al. 1999). Curdlan production was not seen during the cell growth phase and nutrient limitation was required for initiation of curdlan biosynthesis (Lee at al. 1997& Jung et al. 2001). Nitrogen source in the medium is considered as a critical factor in the change of intracellular metabolism, because isoprenoid lipids that play a vital role in carrying cellular oligosaccharides would be more available for curdlan synthesis, instead of cellular lipopolysaccharide synthesis under nitrogen limiting conditions (Lee et al. 1997).

Earlier Kim et al. developed a mutant strain of *Agrobacterium* sp. which produced more curdlan than the wild strain. In this study, we attempted to produce mutant strains through chemical mutagenesis, which could produce curdlan with improvement in yield and productivity than the wild strain. In addition, we optimized downstream process for curdlan recovery and the curdlan was characterized for its purity using various analytical techniques.

## Materials and methods

### Microorganism and mutant development

*Agrobacterium sp*. ATCC 31750 (formerly *Alcaligenes faecalis* subsp. *myxogenes*) and mutants derived from the wild strain were used in this study. Mutant strains that produced more β-glucan were obtained as follows: ATCC 31750 was grown in a 500 ml flask that contained 50 ml of YP medium with shaking at 30°C for 17 h. The YP medium contained (in g/l) sucrose 20, yeast extract 5 and peptone 5, pH 7.0. The cells in 30 ml of broth was washed with 0.1 M citrate buffer, pH 5.5 and suspended in 25 ml of the buffer containing 1 mg/ml of N-methyl-N-nitro-nitrosoguanidine (MNNG). The cells were incubated for 60 min at 30°C, then washed with buffer and cell suspension was spread on agar plates containing 0.05 g/l aniline Blue after appropriate dilution. After incubation for 2 days at 30°C, colonies showing darker blue than the wild strain were isolated for further studies.

### Culture conditions

The seed medium contained (in g/l): sucrose 20, yeast extract 5 and peptone 5, pH 7.0. For flask cultures, the cells (10 ml) grown at 30°C for 17 h in 100 ml of seed medium was inoculated into the fermentation medium that contained (in g/l): sucrose 100, (NH_4_)_2_HPO_4_ 2.3, KH_2_PO_4_ 1, MgSO_4_.7H_2_O 0.4 and 10 ml of trace element solution (5 g FeSO_4_.7H_2_O, 2 g MnSO_4._H_2_O, 1 g CoCl_2_.6H_2_O, 1 g ZnCl_2_ per liter of 0.1 N HCl), 0.3 % (w/v) calcium carbonate. Fermentation was performed in a stirred 7.5 L bioreactor (Bioflo 110, New Brunswick Scientific, USA) equipped with dissolved oxygen (DO) analyzer and a pH controller. Seed culture (300 ml), cultivated at 30°C for 17 h in shake flask was transferred to the fermenter containing 2.7 L of the fermentation medium to initial production in bioreactor. The culture pH was controlled at 7.0 with 4 N NaOH/HCl during cell growth phase and pH was lowered to 5.5 immediately after nitrogen limitation. The aeration rate and the agitation speed were maintained at 1.0 vvm and 700 rpm, respectively.

### Two-step culture

Two-step culture technique was used to study the effect of pH on curdlan production. Cells were grown at 30°C for 17 h in 500 ml baffled flasks containing 100 ml of YP medium on a rotary shaker at 180 rpm. Then the appropriate amount of cells (42–168 mg dry weight harvested by centrifuging at 5000 *g* for 15 min) was transferred to 100 ml of nitrogen free medium for curdlan production. Further cultivation was done at 30°C on a rotary shaker at 180 rpm.

### Analytical methods

One ml of sample was centrifuged at 8000 *g* for 15 min and the supernatant was used to measure sucrose, ammonium and phosphate concentration. The sucrose concentration was measured with a modified dinitrosalicylic (DNS) method. One ml of sample was mixed with 25 μl of 3 M HCl. The mixture was boiled at 100^o^ C for 15 min. After cooling the mixture, 1 ml DNS solution was added and it was boiled at 100°C for 10 min. The absorbance was measured at 540 nm and the sucrose concentration was determined (Miller 1959). For curdlan estimation, one ml of sample with appropriate dilution was mixed with the 15 ml of 3 M NaOH solution and incubated at room temperature for 30 min. The solution was centrifuged at 8000 *g* for 15 min to remove supernatant (curdlan) and pellet (biomass). The supernatant was mixed with 15 ml of 3 M HCl to precipitate curdlan and centrifuged again at 8000 *g* for 15 min. The curdlan was washed three times with distilled water to remove salts. The weight of the curdlan was measured in a weighing dish by drying overnight at 100^o^ C (Lee et al*.*^1999^). Similarly, pellet containing biomass was washed three times with distilled water and dried overnight to measure the cell dry weight. Ammonium concentration was determined by the indophenol method (Srienc et al. 1984). Phosphate concentration was determined with the ascorbic acid method (Chen et al. 1956).

### Molecular characterization of curdlan

#### Monosaccharide composition

Samples were prehydrolyzed with 90 % formic acid under argon in a sealed test tube at 100 °C for 1 h, followed by evaporation of formic acid under a stream of nitrogen. Hydrolysis was then completed using 1 M trifluoroacetic acid (TFA) under argon. After evaporation of TFA, the hydrolysate was dissolved in distilled water and analyzed by thin-layer chromatography (TLC). TLC of carbohydrates was performed using Whatman K-5, 150 Å, silica gel plates with a layer thickness of 250 μm. Monosaccharides were separated using acetonitrile/ water (85:15, v: v) (Robyt and White 1987). Plates were sprayed with sulphuric acid-methanol (1:3, v: v) and heated for 5 min at 110^o^ C to give brown to black spots. The spots obtained with the hydrolysates of sample preparations were compared with standard glucose.

#### Polarimetry

Polysaccharide was dissolved in 1 N NaOH at a concentration of 10 mg/ml and specific rotations and optical rotations were determined using a Perkin-Elmer Polarimeter. Readings were obtained at room temperature using the sodium D-line and a path length of 1 dm (Kenyon and Buller 2002).

#### Aniline blue staining

For sample preparations, 5 ml of aniline blue solution, 0.001 % (w/v) in 50 mM phosphate buffer pH 7.0 was mixed with 50 mg of sample and allowed to stand at room temperature for 15 min. 15 ml of 95 % ethanol was added and polysaccharide was pelleted by centrifugation (10,800 *g* for 10 min at 25^o^ C). After four washes in ethanol, the extent of staining was compared (Nakanishi et al. 1974).

#### Structure analyses

The molecular weight was determined by gel permeation chromatography (GPC). The system consisted of GPC pump (UFLC Shimadzu A20 Shodex OHpak 13u SB-805 HQ 500A Column 600 x 8 mm). 1 mg/ml sample dissolved in 1 N NaOH and DMSO was injected and 0.1 M sodium nitrate was used as solvent. The flow rate was maintained at 1 ml/min. Infrared spectra were obtained on a Fourier transform infrared sprectrometer FT/IR-4200 Jasco, employing potassium bromide (KBr) discs. The transmittance was measured from 450 to 4000 cm^-1^ at a resolution of 4 cm^-1^. ^1^ H and ^13^ C NMR spectra were obtained on Bruker Avance III spectrometer at 500 MHz. The samples were dissolved in deuterated dimethyl sulfoxide at a concentration of 30 mg/ml.

## Results

### Mutant development and curdlan production using shake flask cultures

More than 100 mutants were obtained from Agrobacterium *sp*. ATCC 31750 strain by chemical mutagenesis. N-methyl-N-nitro-nitrosoguanidine (MNNG), a chemical mutagen at a concentration of 1 mg/ml was used to induce mutation. Some strains appeared white and others appeared at different intensities of blue. As reported earlier, only curdlan producing colonies appeared blue on the aniline blue agar plates and also the interaction of the polymer with the dye was proportional to the concentration of the polymer (Nakanishi et al. 1976). Hence we selected the colonies that appeared darker blue compared to the parent strain. Two strains that showed darker blue colour were selected and the curdlan production was evaluated. The strains were sub cultured five times and maintained in agar plates.

**Figure 1:**
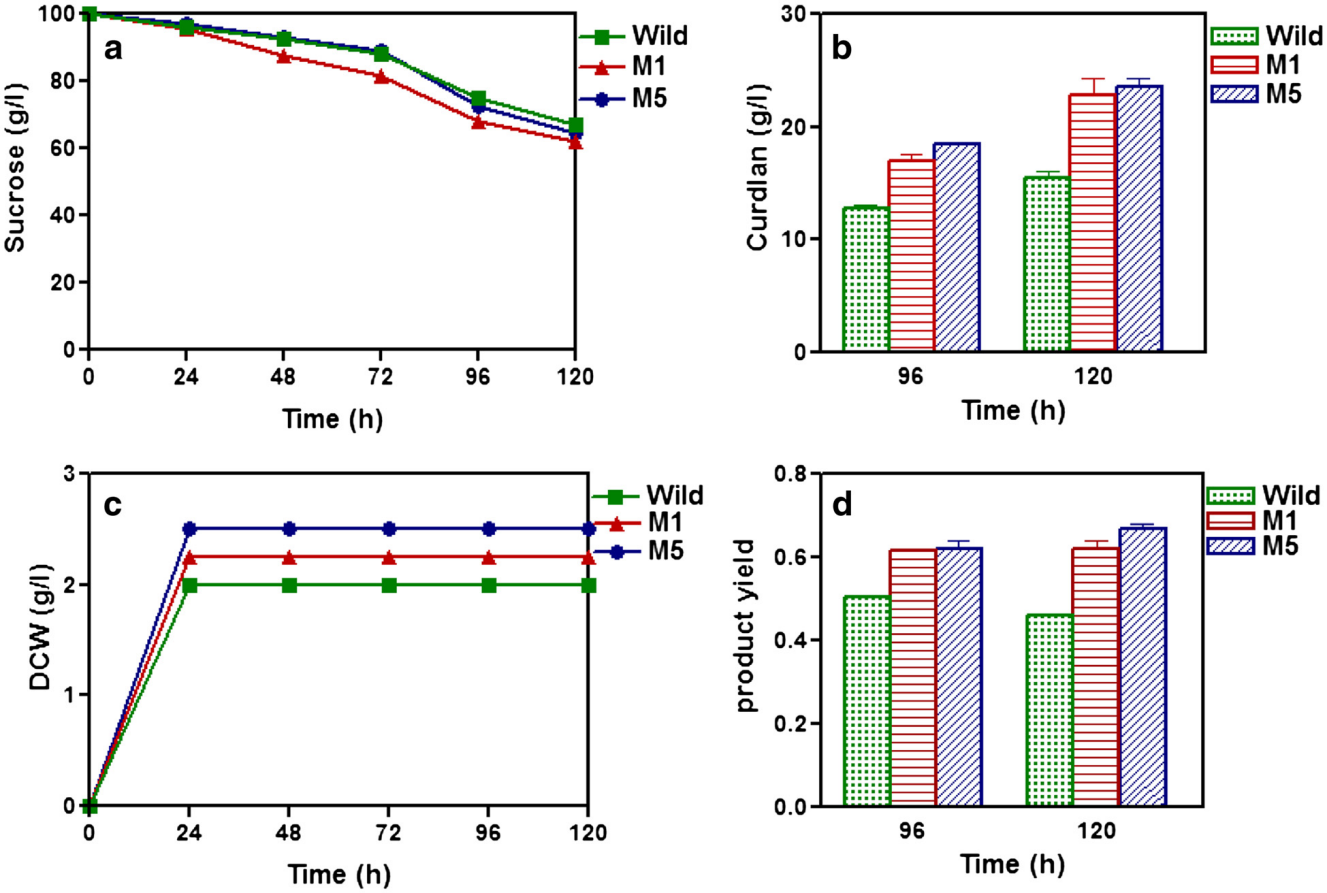
**Curdlan production in shake flask for wild and mutant M1 and M5 strains.** Time course profiles are: a) sucrose, b) curdlan production, c) DCW, and d) product yield for wild, M1 and M5 strains.

Shake flask experiments were carried to compare the mutants with wild strain for β-glucan production. The sucrose utilization profile was almost same for both mutants and wild strains (Figure 1a). The cell concentrations obtained were 2 g/L, 2.5 g/L and 2.75 g/L for wild, M1 and M5 strains respectively (Figure 1c). Maximum curdlan production of 15.5 g/L, 23 g/L and 24 g/L was observed for wild type, M1 and M5 respectively during 96-120 h of cultivation (Figure 1b). The curdlan yield (g β-glucan/g sucrose) for mutant strains M1 and M5 were as high as 0.62 and 0.67 as compared to 0.46 of wild strain (Figure 1d). Further studies were carried out using M5.

Mutant strains showed morphological difference when compared to the wild. They appeared smooth on the agar plate after incubation on agar plate for 24 h at 30°C, while the wild strain appeared rough. The intensity of blue colour shown by the mutants on aniline blue agar was higher than the wild strain. The three strains wild, M1 and M5 were positive for biochemical assimilation tests done with glucose, arabinose, mannose, N-acetyl glucoseamine, maltose, malate and urease. Whereas they showed negative response with citrate, gluconate, adipate and phenyl acetate.

### Curdlan production by *Agrobacterium* sp. in bioreactor

**Figure 2:**
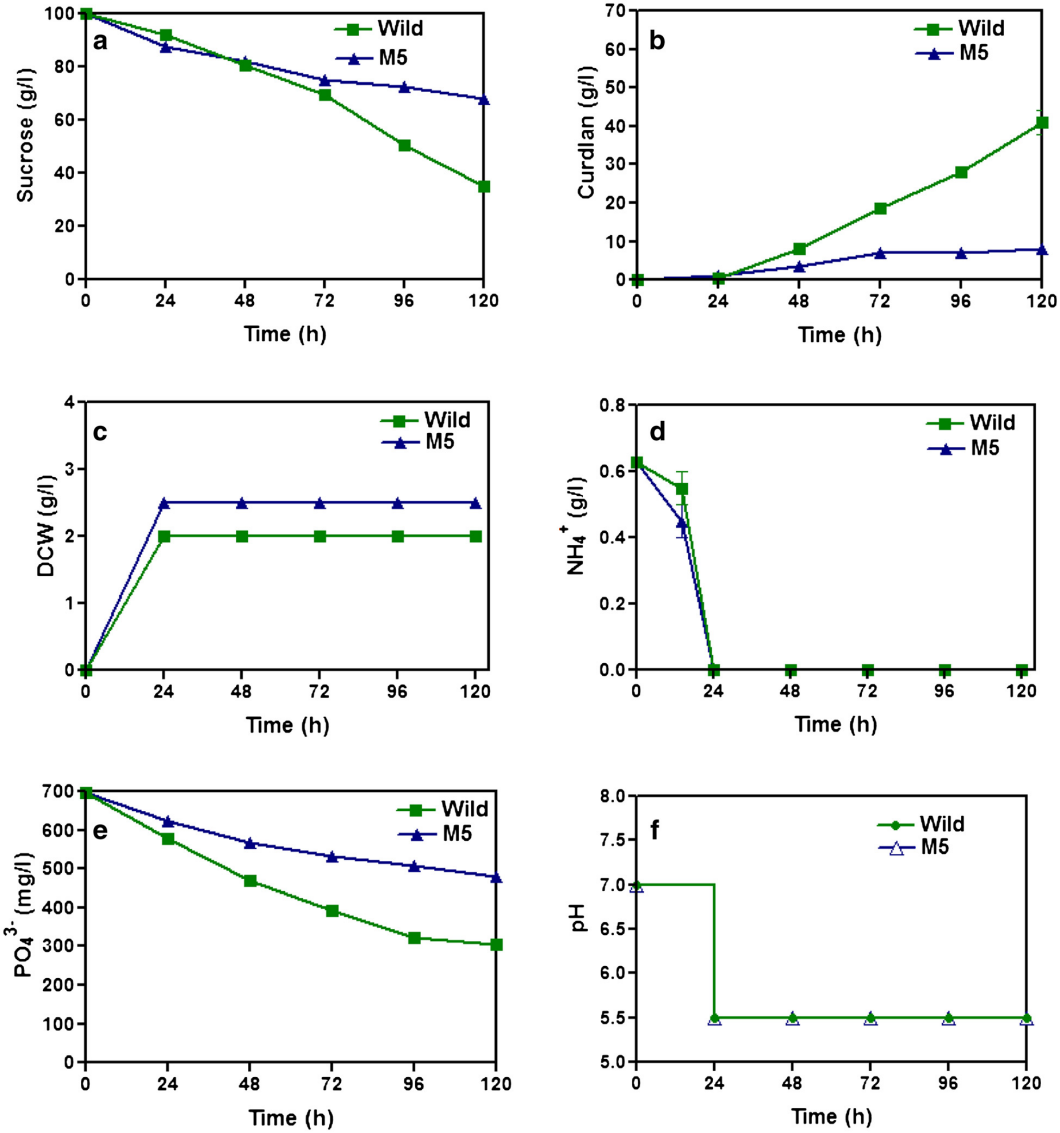
**Production of curdlan by *Agrobacterium *sp. ATCC 31750 wild and mutant M5 strains in a stirred bioreactor with agitation speed 700 rpm and aeration rate 1 vvm, with pH shift after nitrogen limitation.** Time course profiles are: a) sucrose, b) curdlan produced, c) DCW, d) ammonium, e) phosphate and f) pH shift.

Batch fermentation was done to analyse the time profiles of cell growth and curdlan production. As already reported, pH 5.5 was found to be the optimal pH for curdlan production during the fermentation process. The initial concentration of sucrose in the medium was 100 g/L and it was gradually consumed during the course of fermentation and lead to curdlan production. Figure 2a and 2b shows the time courses of sucrose consumption and curdlan production. The wild strain produced a biomass of 2 g/L at the time of nitrogen limitation (Figure 2c). The initial pH of the medium was 7.0 and the pH was shifted to 5.5 after the depletion of ammonium at 24 h (Figure 2f). The ammonium and phosphate consumption profile during the cultivation of *Agrobacterium* species was shown in Figure 2d and 2e. The curdlan production started after nitrogen limitation and reached maximum of 41 g/L in 120 h.

### Curdlan production by M5 strain in bioreactor with pH shift

Production of curdlan by mutant M5 strain in bioreactor was studied and compared with wild strain (Figure 2). The initial concentration of sucrose ammonium and phosphate in the fermentation medium were 100 g/L, 0.627 g/L and 0.698 g/L respectively. The biomass concentration reached 2.5 g/L in 24 h and remained constant after nitrogen depletion in the medium (Figure 2c and d). The pH of the medium was maintained at 7.0 and changed to 5.5 after nitrogen limitation similar to wild strain. Interestingly, it has been found that mutant strain M5 did not utilize sucrose compared to wild strain, which resulted in lower curdlan production (Figure 2a and b). Curdlan production by mutant M5 was 8 g/L which was 80 % lower than that of wild strain (Figure 2b). These results suggest that the conditions for curdlan production by wild strain and mutant M5 were not the same.

### Two step culture to optimize pH for curdlan production in mutant M5 strain

**Figure 3:**
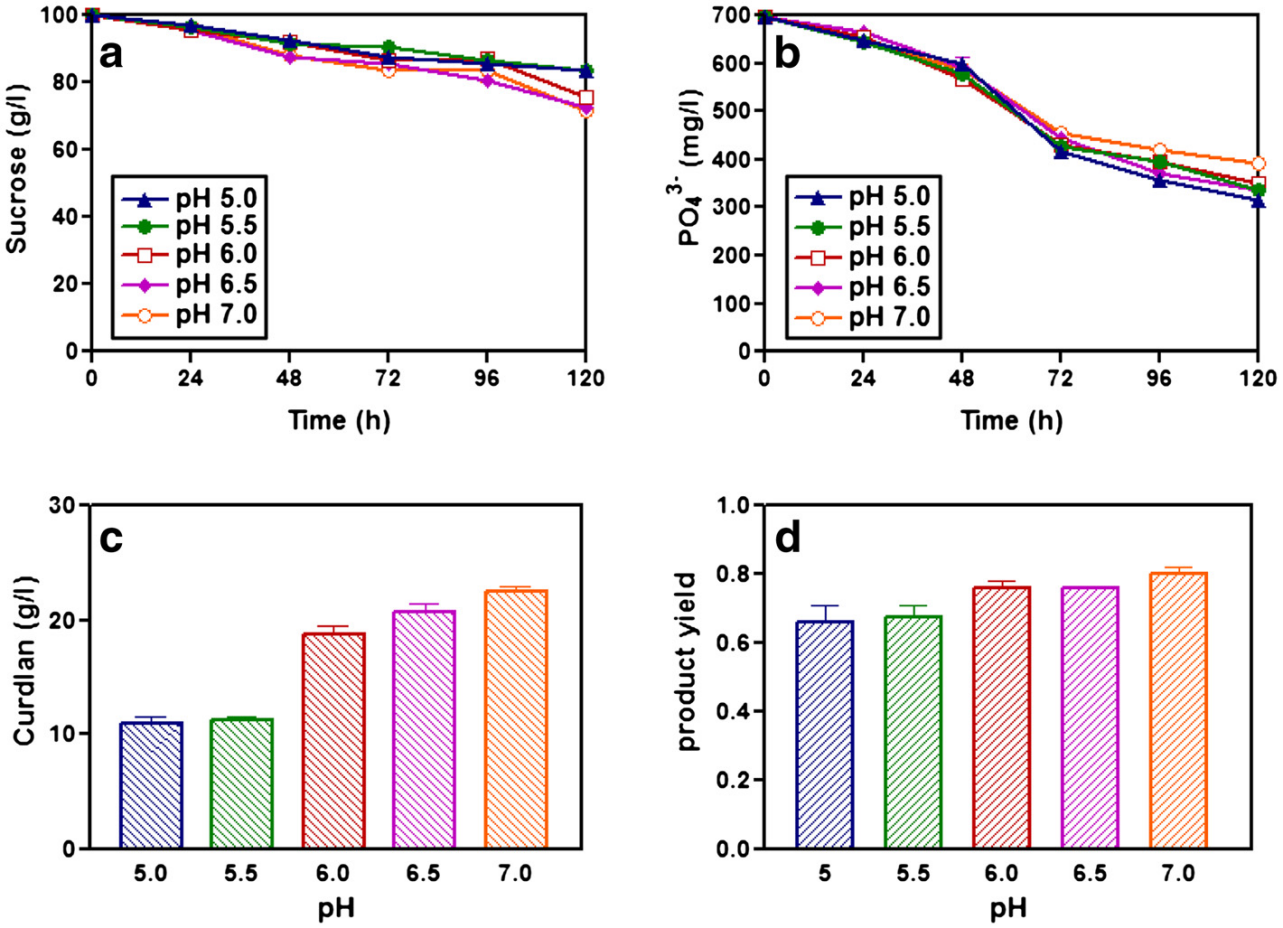
**Effect of pH on curdlan production in nitrogen free medium using two stage culture technique.** Time course profiles are: a) sucrose, b) phosphate, c) curdlan produced and d) product yield of mutant M5 strain in shake flask culture at different pH.

Flask experiments were performed to study the effect of pH on the production of curdlan using M5 strain. Two-step culture technique was employed and the appropriate amount of cells grown in YP medium was transferred to nitrogen free fermentation medium at different pH ranging between 5.0, 5.5, 6.0, 6.5 and 7.0. At pH 5.0, the cells utilized lower amount of sucrose and produced less curdlan (Figure 3a and 3c) compared to the amount of sucrose utilized and curdlan produced at pH 7.0. The strain produced 22.5 g/L curdlan at pH 7.0 which is significantly higher than the amount of curdlan (11 g/L) produced at pH 5.0 during 120 h cultivation. Phosphate was utilized by cells till 72 h and then remained constant for pH ranges tested (Figure 3b). The product yield was higher (0.78) at pH 7.0 when compared to the product yield (0.61) at pH 5.0. These results clearly indicated that optimum pH for curdlan production in mutant M5 was 7.0 and not 5.5 as established for wild strain.

### Curdlan production by M5 strain in bioreactor at constant pH 7.0

**Figure 4:**
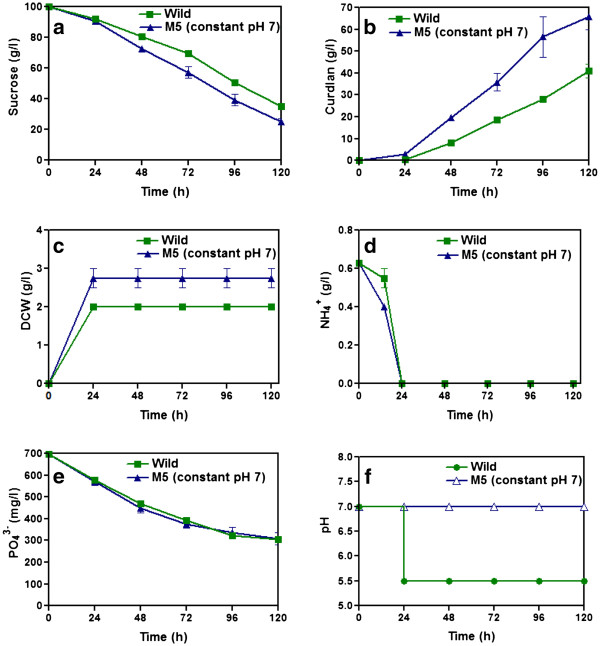
**Production of curdlan by mutant M5 strains in a stirred bioreactor with agitation speed 700 rpm and aeration rate 1 vvm, at constant pH after nitrogen limitation compared with wild strain under pH shift condition.** Time course profiles are: a) sucrose, b) curdlan production, c) DCW, d) ammonium, e) phosphate and f) pH.

Based on two step culture results, bioreactor studies were performed for mutant M5 at constant pH 7.0. Figure 4 shows the time profiles of cell concentration, ammonium, phosphate, sucrose consumption and curdlan production by mutant M5 and compared with wild strain. Nitrogen depletion was achieved within 24 h and 2.75 g/l of cell concentration was achieved (Figure 4c and d). At pH 7.0, the M5 strain consumed sucrose effectively for the production of curdlan. The curdlan production (66 g/l) reached maximum in 120 h which was higher than the wild type (Figure 4b). The sucrose consumption was also higher for M5 strain than the wild strain (Figure 4a). However, the phosphate consumption by M5 strain was same as that of wild strain (Figure 4e). The pH profile for both wild strain and mutant M5 during the culture period is given in Figure 4f.

### Downstream processing of curdlan

**Figure 5:**
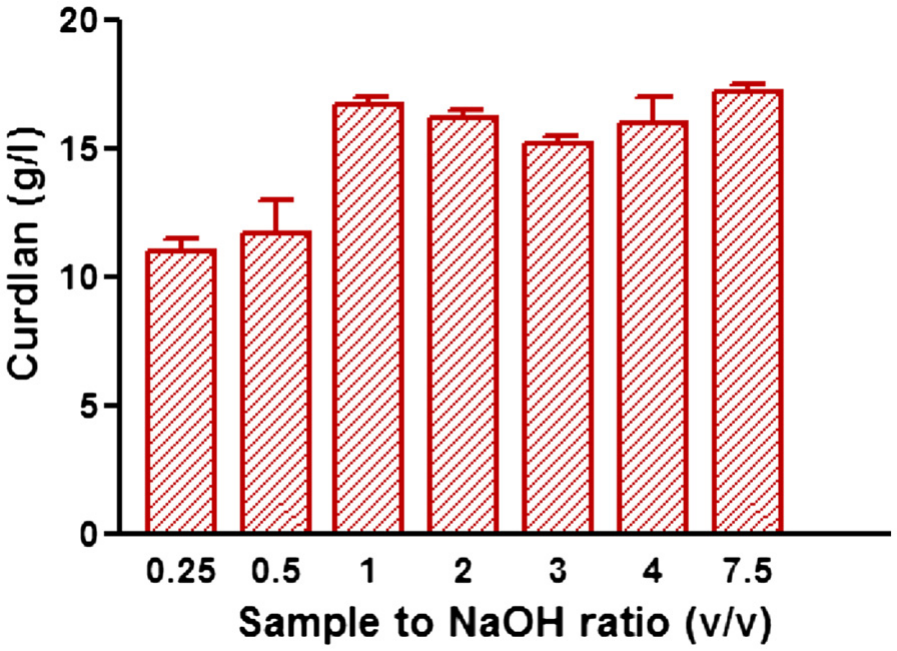
Downstream processing of curdlan with different volumes of 3 N NaOH/HCl.

Curdlan is water insoluble biopolymer but soluble in sodium hydroxide and dimethyl sulfoxide (DMSO). In order to know the optimum amount of NaOH required to extract the curdlan, we varied the ratio of sample volume to 3 N NaOH from 1: 0.25 to 1: 7.5) and checked for the recovery. The curdlan yield was lower when the ratio of sample volume to alkali volume was less than 1:1 (Figure 5). This could be due to insufficient amount of NaOH to dissolve the curdlan from the cells. The curdlan recovery was more or less constant when the ratio of sample volume to alkali volume was 1:1 and above (Figure 5). In earlier reports, the ratio of sample volume to NaOH ratio used was 1: 7.5. Our results clearly showed that even with 1:1 ratio maximum curdlan yield was obtained thereby the cost of downstream processing can be reduced significantly.

### Characterization of curdlan

**Figure 6:**
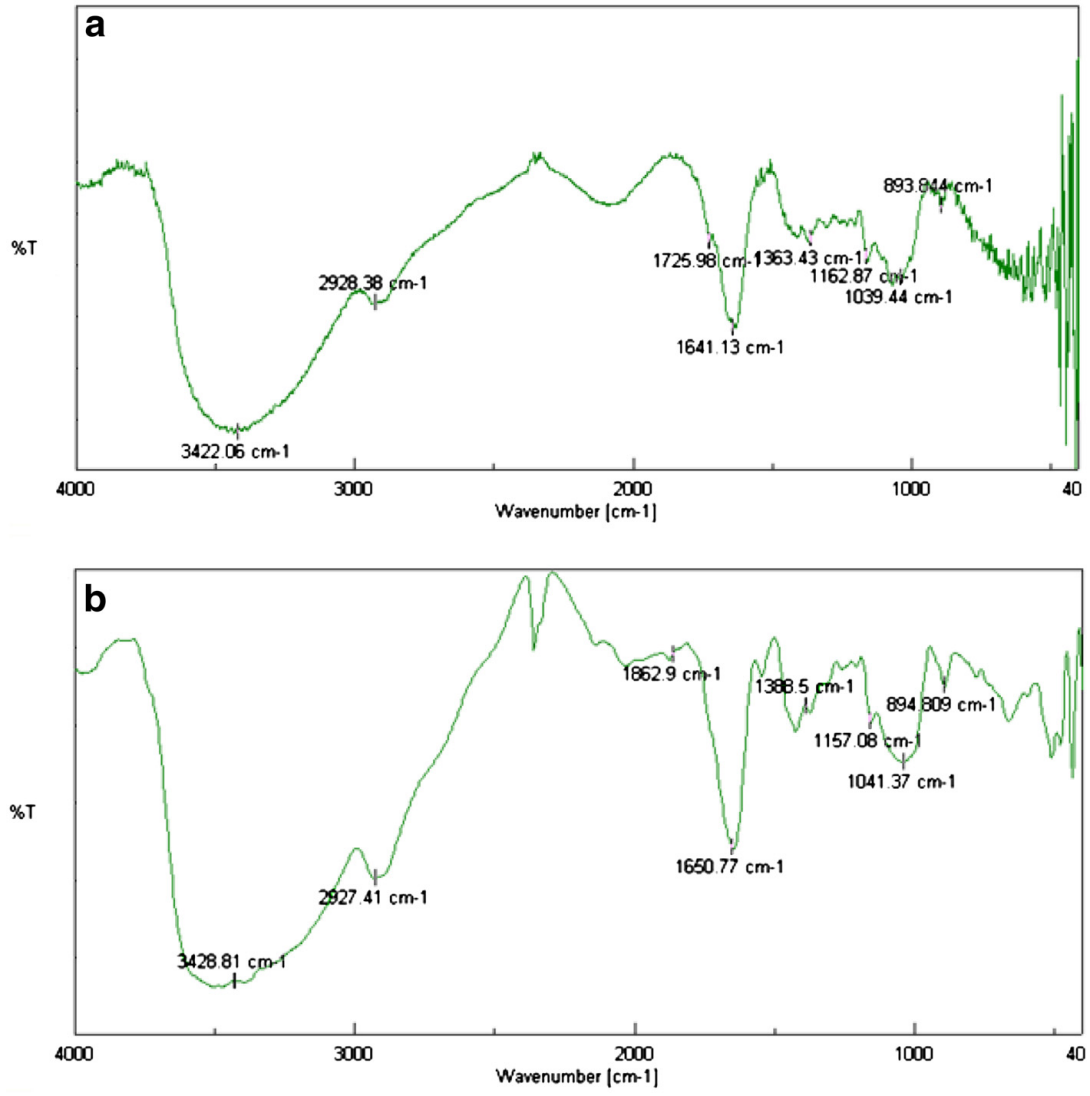
**FTIR spectra of curdlan samples obtained from a wild and b mutant M5 strains of *Agrobacterium *sp. ATCC 31750.**

The specific rotation of curdlan samples obtained from wild, M1 and M5 were +5.2, +1.2 and +3.5 respectively. The optical rotation of curdlan samples obtained from wild, M1 and M5 were +0.052, +0.012 and +0.035 respectively. The low, positive rotation values are indicative of β-linked glucans. The monosaccharide analysis clearly showed that the purified curdlan is exclusively made only of glucose residues as the hydrolysates gave a single spot on TLC. The molecular weight and polydispersity was determined using gel permeation chromatography. The molecular weight of the water insoluble curdlan (dissolved in 1 M NaOH) from the wild and mutant were 6.6 × 10^5^ Da and 5.8 × 10^5^ Da respectively. The polydispersity values were found to be 1.3 and 1.8 for the curdlan obtained from wild and mutant strain respectively. The IR spectra of curdlan obtained from wild and mutant showed absorption band near 890 cm^-1^ (Figure 6a and b) indicative of β-linked glycosidic bonds. It was concluded that no α-configuration existed because there was no characteristic absorption band at 840 cm^-1^. The spectrum of curdlan showed a strong band at 2928.38 cm^-1^, 2927.41 cm^-1^ which is because of C-H stretching. The bend at 1363.43 cm^-1^, 1388.5 cm^-1^ indicated the presence of C-H group and the bend at 1641.13 cm^-1^ and 1650.77 cm^-1^ indicated the presence of C = O group. The bend at 3422.06 cm^-1^ and 3428.81 cm^-1^ was indicative of O-H group (Arli et al. 2011). Identical ^1^ H NMR and ^13^ C NMR spectra were obtained for curdlan samples from wild and mutant strains. The ^1^ H NMR spectrum indicates anomeric protons (4.6-5.2 ppm), sugar protons (3.0-3.9 ppm) and the signal at higher field (δ 4.526) corresponds to β configuration of glucose. The chemical shifts obtained from ^13^ C NMR spectrum were similar to the curdlan spectrum obtained by Saito et al. 1977. The peaks at 103.49 (C-1), 73.31 (C-2), 86.49 (C-3), 68.82 (C-4), 72.82 (C-5) and 61.25 (C-6) ppm are attributed to the signals of the backbone chain for a β- (1, 3)-D glucan.

## Discussion

Different physiological factors are involved in curdlan production by *Agrobacterium* sp. One of the important factors for curdlan production is nitrogen depletion in the medium (Lee et al. 1997& Kim et al. 1999). In this study we isolated a mutant strain having elevated production of curdlan from *Agrobacterium* sp. ATCC 31750 by chemical mutagenesis. The strain developed by us produced 66 g/L curdlan at physiological pH 7.0 which was higher than the curdlan (41 g/L) produced by the wild strain at pH 5.5 after 120 h cultivation. It has been reported that pH 5.5 was optimal for curdlan production (Lee et al. 1999). Initially we attempted to produce curdlan with the mutant strain by following the same strategy that was applied to the wild strain. Interestingly, we obtained contrary results that curdlan production by mutant was lower than the wild strain when pH was shifted to 5.5 after nitrogen limitation (Figure 2a and b). The acidic pH that favoured curdlan production in the wild strain did not favour the mutant strain, as it neither utilized sucrose nor produced curdlan when the pH was shifted from 7.0 to 5.5.

Hence we carried out two stage culture technique to optimize the pH that was favourable for curdlan production in the mutant strain. Two stage culture was most widely used method to study the effect of any variable on production of metabolites (Lee et al. 2001). Our results showed that with increase in pH the cells consumed more sucrose and more curdlan production (Figure 3a and c). We found that the mutant strain produced maximum curdlan (22.5 g/L) and also had a high product yield (0.78) at pH 7.0. Similarly, increase in pH from 5.0 to 7.0 of fermentation medium resulted in increase of polysaccharide yields (Bueno and Cruz, 2006). Production of curdlan at pH 7.0 was studied in bioreactor. At the end of 120 h cultivation, the mutant strain produced 66 g/L curdlan (Figure 4b), which was significantly higher when compared to the wild strain that produced only 41 g/L of curdlan. We found that the product yield with respect to substrate was 0.88 in the mutant which was higher when compared to 0.62 for wild strain. This indicated that the sucrose conversion efficiency was higher in the mutant strain. Interestingly this substrate conversion efficiency happened at normal physiological pH 7.0 in the mutant strain thereby avoiding the pH shift. Thus pH shift after ammonia exhaustion was not required for polysaccharide production in the mutant strain. However, the specific production rate was remained same for both wild and mutant strain. Generally several mechanisms are involved in bacteria that synthesize exopolysaccharides for releasing the polymer from the isoprenoid lipids. A well organised transport system in bacteria ensures that the polymer produced is released from the carrier lipid and transported to the extracellular environment. Sometime mutations could interfere with this process and might lead to internalisation of the polysaccharide (Sutherland 2001). Thus we believe that the mutant strain could effectively utilize sucrose and produce curdlan only at pH 7.0 and at this pH, the mutant strain could effectively transport the polymer produced into the extracellular medium.

To extract curdlan from medium, earlier reports used sample to alkali ratio of at least 1: 7.5 or above (Lee et al. 1999). This will need larger size vessels and the entire downstream processing will be expensive. To reduce the cost, we investigated the exact ratio of sample to alkali and found that the ratio 1:1 is sufficient to extract the curdlan with maximum yield.

The biological activity and the gelling characteristics of the beta glucans mainly depend on their degree of polymerisation, molecular confirmation and molecular weight (Ssaki et al. 1978& Kim et al. 2003). In this study, the curdlan obtained from the wild and mutant had molecular weight of 6.6 × 10^5^ Da and 5.8 × 10^5^ Da respectively. It is already reported that the molecular weight of bacterial curdlan is in the range of 5.3 × 10^4^ Da to 2.0 × 10^6^ Da (Nakata et al. 1998). The results of the monosaccharide analysis confirm that the obtained curdlan is exclusively made only of glucose residues. The presence of peak around 890 nm^-1^ by FTIR shows the β-linkage of curdlan (Zhang et al. 2001& Kim et al. 2003). The proton NMR spectra had peaks indicative of anomeric protons, sugars and β configuration (Wong et al. 2010). Furthermore the peaks obtained in ^13^ C NMR spectra corresponded to the β- (1, 3)-D glucans (Kogan et al. 1988& Saito et al. 1979). Thus a mutant capable of producing more curdlan is isolated in this study. The pattern of curdlan production in the mutant strain varied with the pH of the fermentation medium (Vukojevic et al. 2006) wherein the highest amount of curdlan resulted when the pH of the medium was maintained at 7.0. Hence it can be concluded that, the mutant strain exhibiting elevated curdlan production could be useful in the large-scale production of this commercially important glucan which has got wide application in food and pharmaceutical industries.
